# Enhancing academic writing skills and motivation: assessing the efficacy of ChatGPT in AI-assisted language learning for EFL students

**DOI:** 10.3389/fpsyg.2023.1260843

**Published:** 2023-12-15

**Authors:** Cuiping Song, Yanping Song

**Affiliations:** ^1^School of Foreign Studies, North Minzu University, Yinchuan, Ningxia, China; ^2^School of Public Administration, Central South University, Changsha, Hunan, China

**Keywords:** AI-assisted language learning, writing skills, writing motivation, EFL students, mixed methods study, ChatGPT

## Abstract

**Introduction:**

This mixed-methods study evaluates the impact of AI-assisted language learning on Chinese English as a Foreign Language (EFL) students’ writing skills and writing motivation. As artificial intelligence (AI) becomes more prevalent in educational settings, understanding its effects on language learning outcomes is crucial.

**Methods:**

The study employs a comprehensive approach, combining quantitative and qualitative methods. The quantitative phase utilizes a pre-test and post-test design to assess writing skills. Fifty EFL students, matched for proficiency, are randomly assigned to experimental (AI-assisted instruction via ChatGPT) or control (traditional instruction) groups. Writing samples are evaluated using established scoring rubrics. Concurrently, semi-structured interviews are conducted with a subset of participants to explore writing motivation and experiences with AI-assisted learning.

**Results:**

Quantitative analysis reveals significant improvements in both writing skills and motivation among students who received AI-assisted instruction compared to the control group. The experimental group demonstrates enhanced proficiency in various aspects of writing, including organization, coherence, grammar, and vocabulary. Qualitative findings showcase diverse perspectives, ranging from recognition of AI’s innovative instructional role and its positive influence on writing skills and motivation to concerns about contextual accuracy and over-reliance. Participants also reflect on the long-term impact and sustainability of AI-assisted instruction, emphasizing the need for ongoing development and adaptation of AI tools.

**Discussion:**

The nuanced findings offer a comprehensive understanding of AI’s transformative potential in education. These insights have practical implications for practitioners and researchers, emphasizing the benefits, challenges, and the evolving nature of AI’s role in language instruction.

## 1 Introduction

Academic writing holds a pivotal role in the language development of English language learners, necessitating proficiency in diverse areas such as writing organization, coherence, grammar, and vocabulary (Campbell, 2019). Proficient writing skills empower learners to effectively communicate their ideas, articulate thoughts clearly, and achieve academic excellence across various professional domains (Yoon, 2011). However, the process of monitoring and providing insightful feedback on student writing poses challenges in terms of time, effort, and subjectivity (Yu and Lee, 2014; Lim and Phua, 2019). Moreover, English language learners often face motivation constraints due to time limitations, which hinders their ability to allocate sufficient time and effort toward improving their writing abilities (Lee, 2017).

The integration of technology in English language classrooms is widely recognized as a means to overcome certain obstacles in language learning processes (Roll and Wylie, 2016; Knox, 2020), particularly in writing tasks where time constraints often arise (Stapleton and Radia, 2010; Kessler, 2020; Rahimi and Fathi, 2022; Wang, 2022). With the ubiquitous availability of technology and online platforms, learners now have the convenience of practicing their language skills, specifically writing, at any time and from anywhere (Yan, 2023). This includes the use of advanced artificial intelligence (AI)-based computer and mobile programs, which offer interactive and personalized tools for honing writing abilities as well as enhanced motivation (Jiang, 2022; Meunier et al., 2022; Yan, 2023).

The emergence of AI-powered writing tools, accessible on mobile devices, provides a novel avenue to address the challenges associated with developing writing proficiency through traditional training methods (Zawacki-Richter et al., 2019; Jia et al., 2022; Kohnke, 2023). These AI-assisted writing tools offer automated feedback on various aspects of writing, including organization, coherence, grammar, and vocabulary, thereby facilitating more effective writing performance improvements. Additionally, learners can expedite their writing development as AI tools help them identify and rectify grammatical and lexical errors, while also suggesting alternative sentence structures to enhance the overall writing quality and structure (Zhao, 2022; Chen, 2023; Salvagno et al., 2023).

Numerous research studies have explored the effects of AI on enhancing English language learning outcomes (Sun et al., 2021; Huang A. Y. et al., 2023). Huang X. et al. (2023), for example, investigated the academic performance and level of engagement among students who participated in an AI course compared to those in a non-AI course. The findings revealed that students in the AI course exhibited superior academic achievement and demonstrated higher levels of active participation in their learning tasks compared to their counterparts in the non-AI course. Additionally, several studies have specifically focused on the impact of AI-assisted language learning tools in improving English language learners’ writing skills (Liu et al., 2021; Seufert et al., 2021; Wu et al., 2021; Fitria, 2023; Hsiao and Chang, 2023; Yan, 2023). For instance, Liu et al. (2021) examined the influence of AI-supported language learning on the writing skills of English as a Foreign Language (EFL) learners and found that the AI-supported approach had a significant positive impact on their writing abilities. Similarly, Yan (2023) investigated the impact of ChatGPT, an AI-assisted language learning tool, on the writing skills of EFL learners and reported significant improvements in their writing performance as a result of AI-assisted language learning.

Within the domain of English language learning, a conspicuous research gap emerges, one that delves into the intricate role of AI-assisted language learning tools in enhancing the writing proficiency and motivational drive of English language learners, notably within the unique context of EFL. This research gap assumes heightened significance in light of the pervasive challenges faced by EFL learners, often struggling to allocate sufficient time for refining their writing skills, consequently hampering their overall writing competence (Fathi and Rahimi, 2022). Hence, this study embarks on a journey to meticulously investigate the precise impact that AI-assisted language learning exerts on the writing prowess and motivational outlook of Chinese EFL learners. Furthermore, the research undertakes a comprehensive exploration of learners’ perceptions and assessments regarding the effectiveness of AI-assisted language learning instruction, thus enhancing the depth and breadth of the research findings.

The outcomes of this study bear paramount significance for both EFL educators and pedagogical methodologies alike. Notably, the findings furnish compelling evidence in favor of the integration of AI-assisted writing tools within EFL classrooms, illustrating a substantial augmentation in students’ proficiency in written expression. Furthermore, by endorsing collaborative writing endeavors facilitated by AI-powered tools, educators can cultivate and sustain students’ enthusiasm and vested interest in the writing process, thus nurturing their overall growth and proficiency in writing.

## 2 Literature review

### 2.1 Theoretical framework

This study is underpinned by Vygotsky's (1984) social constructivism, which underscores the pivotal role of social interaction in the learning process. Vygotsky contends that learning transpires through the dynamic interplay between individuals and their social milieu, especially in collaborative endeavors with more knowledgeable peers. He posits that cultural development manifests initially in social interactions, subsequently becoming internalized at an individual level. At the core of Vygotsky’s social constructivism lies the concept of the Zone of Proximal Development (ZPD), representing the space between a learner’s current level of independent problem-solving and their potential for further advancement through collaborative problem-solving with more capable peers. Engaging in collaborative language learning and seeking assistance from others enable learners to effectively reach their ZPD, showcasing their ability to work independently within the learning context as indicative of ZPD mastery.

Kim (2008) elucidates that a group of learners can mutually support each other in reaching their ZPD, alternating roles as both less and more skilled learners contingent on the language learning tasks and activities. In essence, through group or pair work in various language-learning tasks, learners amalgamate their diverse language-learning proficiencies and knowledge, thus assisting each other in achieving their ZPD (Oxford, 1997). While traditional interpretations of social constructivism emphasize human interactions, we extend this framework to probe into the potential of AI-assisted language learning in emulating social interactions and collaborative learning experiences.

In the EFL education, students often derive substantial benefits from peer collaboration and social interactions in augmenting their language and writing skills (Fathi et al., 2020). Vygotsky’s theory underscores the significance of the ZPD, where learners can make strides with the guidance and collaboration of more knowledgeable peers. AI, exemplified by ChatGPT, holds the promise of fabricating an environment mirroring these collaborative interactions, by providing real-time feedback, suggestions, and support akin to peer assistance. By employing AI-assisted language learning tools, learners not only partake in social interactions but also engage in dynamic exchanges with AI, effectively treating it as a knowledgeable and adaptive virtual peer. While AI is conventionally viewed as an individualized learning tool, our inquiry delves into its potential to contribute to writing skill development and motivation by nurturing a sense of collaboration and social engagement among learners. This amalgamation of AI and social constructivism is vital as it enables an exploration of the potential synergy between technological advancements and entrenched learning theories. By harnessing the capabilities of AI, learners can receive immediate, personalized feedback, access a wealth of linguistic resources, and customize their learning journey to their specific needs and inclinations. This reciprocal interplay between human learners and AI amplifies the collaborative learning milieu, potentially enhancing the effectiveness of language acquisition and writing skill development.

Importantly, feedback is recognized as a critical component in enhancing writing performance and motivation in the context of language learning (Bakla, 2020; Liu et al., 2022; Zhang and Zou, 2023). While Vygotsky’s theory provides a foundation for understanding collaborative learning, it is essential to acknowledge the substantial body of research that emphasizes the role of feedback in shaping writing proficiency and motivation. Feedback, whether from human instructors or AI systems, plays a central role in guiding learners toward improvement (Loncar et al., 2023; Zhang and Zou, 2023). In traditional approaches, teacher feedback has been a cornerstone of writing instruction, offering valuable insights into areas for improvement. Similarly, in AI-assisted learning, ChatGPT’s real-time feedback mechanisms provide learners with continuous guidance and suggestions for enhancing their writing skills. Recognizing the interplay between feedback and collaborative learning is crucial in assessing the effectiveness of AI-assisted language learning.

While Vygotsky’s theory is primarily associated with collaborative learning and scaffolding, it also holds relevance for understanding key constructs like engagement, self-regulation, and personalized learning within the context of AI-assisted language learning (Hadwin and Oshige, 2011; Schrader, 2015; Kucirkova and Littleton, 2017). Engagement, as a crucial aspect of the learning process, is influenced by the dynamic interaction between learners and their learning environment (Fredricks et al., 2016). In the context of AI-assisted instruction, engagement takes on a unique dimension. The interactive nature of AI tools, such as ChatGPT, allows learners to actively participate in the writing process, seeking immediate feedback and refining their writing skills in real-time. This heightened engagement, facilitated by the AI tool’s responsiveness, contributes to the observed improvements in writing skills and motivation (Liu et al., 2021; Utami and Winarni, 2023).

Furthermore, Vygotsky’s constructivist perspective sheds light on the concept of self-regulation in the context of AI-assisted learning. Through collaborative writing activities with the AI, learners transition from being externally regulated to becoming self-regulated writers. The AI tool acts as a facilitator, guiding learners to internalize effective writing strategies and enabling them to complete tasks independently (Zimmerman, 2002). This transition to self-regulation is a critical component of the learning process and is essential for long-term skill development.

Additionally, the personalized learning experience facilitated by AI tools aligns with the principles of Vygotsky’s social constructivism. Learners have the opportunity to work at their own pace and receive immediate, tailored feedback, allowing for a more individualized learning journey (Huang A. Y. et al., 2023; Huang X. et al., 2023). This aligns with Vygotsky’s idea that learning occurs most effectively when it is situated within a learner’s ZPD, the gap between their current level of competence and their potential for development with guidance. In this sense, the AI tool functions as a scaffold, helping learners reach their ZPD and gradually internalize writing skills. This personalization contributes to the observed enhancements in writing skills and motivation (Fulton et al., 2021). In extending Vygotsky’s social constructivist framework, this study advances our comprehension of collaborative learning by scrutinizing the symbiotic relationship between human learners and AI-driven virtual peers. Through this innovative fusion, we aspire to unlock new vistas for ameliorating academic writing skills and motivation among EFL students.

### 2.2 Artificial intelligence

AI can be defined as a system incorporating intelligent programs that collaborate with humans to perform various tasks (Aldosari, 2020). In educational settings, AI has the capability to make intelligent decisions akin to human decision-making (Akerkar, 2014). Applied linguistics researchers have recognized the potential of AI in language learning and teaching contexts, aiming to enhance teaching methodologies for language instructors and facilitate language learners’ language acquisition (Luckin et al., 2016; Zhang and Zou, 2020; Nazari et al., 2021; Sun et al., 2021; Xia et al., 2022). AI-assisted online platforms can be employed to generate the necessary language input and output, aiding language learners in their language development. These AI tools, accessible on computer and mobile devices, particularly support the enhancement of writing skills. One noteworthy AI-powered tool is ChatGPT, an AI-assisted Chatbot created by OpenAI (Barrot, 2023). ChatGPT can be effectively utilized in diverse language learning courses to enhance learners’ writing abilities (Barrot, 2023). Equipped with comprehensive knowledge, ChatGPT generates words and grammatically correct structures to facilitate the creation of coherent and cohesive written text. This tool comprehends human queries and provides appropriate responses. Moreover, ChatGPT assists language learners in addressing writing challenges related to organization, coherence, grammar, and vocabulary. It offers alternative suggestions to rectify ungrammatical sentences and improve overall writing proficiency.

ChatGPT has been recognized for its potential in enhancing writing performance (Huang and Tan, 2023). The AI-powered tool facilitates the production of coherent and cohesive text by providing learners with immediate feedback and alternative grammatically correct sentences (Huang and Tan, 2023). However, it is important to consider certain limitations when using ChatGPT for different writing tasks. Frequent reliance on generated text from ChatGPT may hinder language learners’ own writing abilities. Additionally, using the generated text without appropriate review and editing may lead to issues of plagiarism that should be carefully addressed (Huang and Tan, 2023).

Numerous studies have investigated the positive impact of AI-assisted language learning tools on English language learners’ language acquisition skills (Suryana et al., 2020; Divekar et al., 2021; Liu, 2021; Bašić et al., 2023; Bishop, 2023; Fitria, 2023). For instance, Rahman et al. (2022) examined the role of an AI-assisted language learning tool in identifying and addressing grammatical errors, leading to the development of writing skills among EFL learners. The results indicated significant improvement in the EFL learners’ writing skills, and the learners themselves expressed positive perceptions regarding the effects of AI-assisted language learning on their writing abilities. Utami and Winarni (2023) conducted a case study research on three Indonesian EFL learners, exploring their use of AI-assisted language learning for academic research writing. Through a combination of quantitative data collected via questionnaires and qualitative data obtained through interviews, the findings revealed that AI-assisted language learning tools positively contributed to the learners’ academic research writing and increased their engagement in such tasks.

Similarly, Seufert et al. (2021) investigated the effects of instructor and peer feedback integrated with intelligent tutoring systems feedback on the academic writing skills of EFL students. The intelligent tutoring system’s feedback, provided within an AI-assisted language learning environment, had a significant impact on the development of students’ academic writing skills. Hwang et al. (2023) conducted an experimental study with a control group to explore the effects of an AI-based writing feedback tool on undergraduate EFL students’ writing performance. The results demonstrated that the experimental group, utilizing the AI-assisted tool, outperformed the control group in writing tasks. The personalization feature of the AI tool played a crucial role in facilitating learners’ revision and editing of their writing assignments.

Fitria (2021) conducted a study that investigated the effectiveness of Grammarly as an AI-assisted language learning tool in enhancing the writing performance of EFL learners. The learners received corrective feedback within the AI-powered environment to revise and improve their written texts. The findings revealed a significant contribution of the AI-assisted language learning tool to the improvement of learners’ writing skills. Similarly, Chang et al. (2021) employed a quasi-experimental research design to examine the impact of an AI-supported writing feedback tool on EFL learners’ writing performance. The experimental group utilized Grammarly for editing and revising their written texts, while the control group did not have access to Grammarly. The results demonstrated that the experimental group outperformed the control group in writing skills, highlighting the significant role of this AI-powered language learning tool in developing EFL learners’ writing performance.

Gayed et al. (2022) developed an AI-assisted language learning program to address cognitive barriers and improve the writing performance of EFL students. The findings indicated that the AI-powered language learning tool effectively enhanced students’ writing performance and reduced cognitive barriers encountered during writing tasks. In a study by Yan (2023), the contribution of ChatGPT as an AI-powered language learning tool to EFL learners’ English writing was explored. The study examined learners’ reactions and reflections on the use of the AI-assisted tool to develop their academic writing performance. The results indicated a significant role of the AI tool in improving learners’ writing performance and enhancing their efficiency in completing writing tasks. However, learners expressed concerns about potential negative effects on their academic writing skills over time and emphasized the need for instruction on the appropriate application of the tool in their academic writing tasks. Su et al. (2023) asserted that the integration of ChatGPT in language learners’ writing courses proved beneficial for accomplishing argumentative writing tasks. They emphasized the linguistic and structural complexities involved in such tasks, making it challenging for peers or instructors to provide effective feedback in interactive writing activities. To address this, ChatGPT was introduced to provide more efficient feedback and comments on language usage, organization, and content, significantly enhancing learners’ argumentative writing tasks. Similarly, Ippolito et al. (2022) utilized Wordcraft, an AI-powered text editor, to support professional writers in creative writing tasks, particularly in brainstorming. The results indicated the positive impact of the AI-powered tool on creative writing.

Abdullayeva and Musayeva (2023) examined the influence of ChatGPT on EFL learners’ writing skills and found that it contributed by providing writing prompts, immediate feedback, and revision suggestions. Nazari et al. (2021) conducted a true experimental study investigating the effects of AI-assisted language learning on EFL learners’ writing performance. The findings revealed that learners who utilized the AI-powered tool outperformed those who did not in terms of writing performance. Additionally, the AI learners displayed high engagement behaviorally, cognitively, and emotionally in AI-supported writing activities.

In a quasi-experimental research design, Liu et al. (2021) explored the impact of AI on EFL learners’ writing skills. The findings indicated substantial improvements in writing skills compared to the conventional class. The AI-supported language learning approach also enhanced learners’ self-efficacy, self-regulated learning, and reduced cognitive load, contributing to their effective writing performance.

Taken together, in reviewing the extensive body of literature on AI-assisted language learning, numerous studies have made valuable contributions to our understanding of its impact on language acquisition and the broader field of language learning (e.g., Suryana et al., 2020; Divekar et al., 2021; Liu, 2021; Bašić et al., 2023; Bishop, 2023; Fitria, 2023). These studies have provided critical insights into various aspects of language learning, including vocabulary acquisition, grammar correction, and language proficiency improvements. However, as we delve deeper into the literature, it becomes evident that a significant gap persists in terms of the specific influence of AI on the development of academic writing skills and writing motivation in EFL learners.

While a select few studies, such as those by Rahman et al. (2022) and Fitria (2021), have ventured into the realm of writing skills, the scope has remained limited to the correction of grammatical errors and broader language proficiency (e.g., Liu et al., 2021; Gayed et al., 2022). These studies, though informative, have not comprehensively addressed the nuances of academic writing or the intricate factors that contribute to writing motivation. Moreover, there is an absence of research that holistically investigates the combined impact of AI tools on both academic writing skills and motivation.

This study seeks to address this significant gap by embarking on a more focused examination of AI-assisted language learning, with a particular emphasis on academic writing skills and writing motivation. While our predecessors have laid a foundation for AI’s role in writing skill development, we aim to extend this foundation by delving into the intricate processes that underlie academic writing and the dynamics of motivation in the EFL context. Through this research, we aspire to provide a more in-depth understanding of the multifaceted role of AI in the realm of academic writing, an area that has been insufficiently explored in the existing literature.

Our research not only evaluates the quantitative impact but also ventures into the qualitative aspects of EFL learners’ interactions with AI in academic writing activities. By doing so, we aim to uncover the subtleties and complexities that underlie learners’ perceptions, attitudes, and experiences, contributing to a comprehensive picture of the influence of AI on both academic writing skills and motivation. The insights garnered from this study have the potential to significantly inform and enrich AI-assisted language learning practices, particularly in the context of EFL education, where tailored solutions are essential for addressing the unique challenges faced by learners, such as varying language proficiency levels, time constraints, and motivation fluctuations.

### 2.3 Purpose of the study

The existing literature review underscores the substantial impact of AI-assisted language learning tools on improving various facets of writing skills and engagement among English language learners (Nazari et al., 2021; Abdullayeva and Musayeva, 2023; Su et al., 2023; Utami and Winarni, 2023; Yan, 2023). This body of research highlights the positive perceptions of English language learners regarding the integration of AI tools into writing activities (Rahman et al., 2022; Utami and Winarni, 2023). While these findings have significantly contributed to our understanding of the potential benefits of AI in language learning, several factors underscore the need for a more focused investigation.

First, despite the increasing adoption of AI in language learning, there is a distinct gap in research that delves into the specific effects of AI-assisted language learning on academic writing skills and writing motivation. Academic writing is a critical skill for EFL learners, and understanding how AI tools can enhance this skill is of paramount importance for both educators and learners. Second, within the context of EFL education, where learners often face unique challenges related to language proficiency, time constraints, and motivation, there is a need for tailored solutions. This study aims to fill this void by exploring the impact of AI-assisted language learning tools specifically within the EFL context, providing insights that can inform pedagogical practices and curriculum design. Third, while existing studies have highlighted the positive perceptions of learners regarding AI tools, a more nuanced understanding of their perceptions, attitudes, and experiences is essential. This research not only examines the quantitative impact but also delves into the qualitative aspects of EFL learners’ interactions with AI in academic writing activities.

In light of these factors and the evolving landscape of language education, this study seeks to address this multifaceted knowledge gap by thoroughly examining how AI-assisted language learning influences the academic writing skills, writing motivation, and perceptions of EFL learners. By doing so, it aims to provide valuable insights into the role of AI in improving language proficiency and pedagogical practices within the context of EFL education. To address these research objectives, the following research questions were formulated:

1. To what extent does AI-assisted language learning instruction contribute to the enhancement of academic writing skills in EFL learners compared to non-AI-assisted instruction?
2. To what extent does AI-assisted language learning instruction contribute to the enhancement of writing motivation in EFL learners compared to non-AI-assisted instruction?
3. What are the perceptions of EFL learners toward the impact of AI-assisted language learning on their academic writing skills and writing motivation?

## 3 Method

### 3.1 Participants

The study included 50 Chinese EFL students who were enrolled in a Bachelor’s degree program at a national university in China. Participants were recruited through campus bulletin board announcements and email invitations sent to eligible individuals who met specific inclusion criteria. To be included in the study, participants had to be enrolled in the university’s English language program and have at least two years of prior English language instruction. They could not have previously participated in any AI-assisted language learning programs. Prior to their involvement, participants provided written informed consent after receiving detailed explanations of the study’s purpose, procedures, potential risks, and benefits. They were assured of their right to withdraw from the study at any time without penalty.

A multistage sampling procedure was employed to ensure a representative sample. The university’s English language program department generated a list of eligible participants, from which a random sample of students was selected using a computer-generated random number method. The selected students were then invited to participate in the study. Random assignment was used to assign participants to either the experimental group or the control group. This randomization process aimed to minimize bias and ensure comparability between the groups, giving each participant an equal chance of being assigned to either group. The study was conducted during the regular academic year at the university, utilizing the language laboratories and computer facilities provided by the institution. Participants’ regular English language classes were unaffected, ensuring they received the standard curriculum and instruction during the study period.

To establish a comparable proficiency level, an English proficiency test was administered as part of the screening process. This comprehensive test assessed participants’ reading, writing, listening, and speaking skills, confirming their similar level of English proficiency confirming their intermediate to upper-intermediate level of English proficiency. The participants came from diverse backgrounds, representing various regions of China. On average, they had been studying English as a foreign language for five years. Their ages ranged from 18 to 22 years old, with the majority being in their second or third year of undergraduate studies. Participants pursued majors in disciplines such as engineering, business, social sciences, and humanities. Throughout the study, confidentiality and anonymization measures were in place to protect participants’ privacy. They were informed about these procedures and the steps taken to ensure the security of their data. Over a three-month period, both the experimental and control groups were taught by a male experienced teacher who used the same instructional materials for both groups.

### 3.2 Instruments

#### 3.2.1 Writing skills assessment

The study utilized the International English Language Testing System (IELTS) academic writing tasks 1 and 2 as pre-tests and post-tests to evaluate participants’ academic writing skills. The selection of writing tasks was based on reputable resources commonly employed in IELTS preparation. These resources were chosen to ensure alignment with the instructional content provided to both groups. The participants’ writing proficiency was assessed using the IELTS writing band descriptors for task 1 and task 2 (see Appendix A), which encompassed criteria such as task achievement, coherence and cohesion, lexicon, and grammatical range and accuracy (University of Cambridge ESOL Examinations, 2011). To enhance objectivity in the scoring process, two independent raters evaluated the writing samples for the pre-tests and post-tests. The raters consisted of the researcher/instructor (second author) and an experienced IELTS instructor who had received official training at an IELTS training center. Both raters possessed the necessary expertise to accurately assess the participants’ academic writing skills. Inter-rater reliability between the two raters was assessed, demonstrating a high level of agreement with a correlation coefficient of 0.88.

#### 3.2.2 Writing motivation scale questionnaire

In order to examine any changes in participants’ motivation following the implementation of the AI-assisted instruction, the study utilized the Writing Motivation Scale (WMS) which is a well-established tool in social science research for gathering data on various social aspects, behaviors, attitudes, and underlying reasons for actions (Fathi et al., 2023). The adapted version of the WMS used in this study (see Appendix B) was based on the original scale developed by Waller and Papi (2017). The questionnaire consisted of seven items that assessed students’ intended efforts for learning the second/foreign language, motivation to learn the second/foreign language, motivational intensity, teacher feedback, content and organization of the course, and peer feedback. Participants rated each item on a five-point Likert scale ranging from 1 (never) to 5 (always). The internal consistency of the scale was evaluated using Cronbach’s Alpha and was found to be satisfactory in this study (α = 0.86).

#### 3.2.3 Semi-structured interview

Semi-structured interviews (see Appendix C) were conducted with nine EFL students from the experimental classroom group to gain in-depth insights into the AI-assisted instruction. The interviews aimed to capture diverse perspectives by including participants of different genders and writing performance levels (low, mid, and high performers). The interviews were conducted in the participants’ native language to ensure clear expression of ideas and attitudes toward the course. Pseudonyms (S1, S2, etc.) were used to maintain confidentiality and adhere to ethical guidelines. Each interview lasted approximately 30 min and began with rapport-building questions. Participants were then encouraged to discuss their attitudes and perceptions toward the AI-assisted instruction, compare it to conventional instruction, and identify any advantages, disadvantages, and challenges encountered. Audio recordings of the interviews were transcribed and translated into English for analysis. To ensure credibility, a member-checking technique was employed, where participants reviewed and verified the transcriptions. Data analysis involved multiple readings of the transcripts to identify key ideas and themes.

### 3.3 Procedure

#### 3.3.1 Experimental group

The experimental group in this study received AI-assisted writing instruction using ChatGPT, an advanced language model. Participants in this group accessed a web-based interface specifically designed for the study and were instructed on how to effectively interact with ChatGPT to enhance their writing skills. These students had the flexibility to use ChatGPT both at home and in the classroom. They were encouraged to engage with the AI-assisted writing tool at their convenience, allowing for a personalized learning experience tailored to their schedules and preferences. This approach ensured that participants had regular exposure to AI assistance throughout the 12-week intervention period.

During the intervention, participants logged into the platform and selected a combination of classroom exercises and topics of interest to them. As they wrote their responses, ChatGPT provided real-time feedback on grammar, vocabulary usage, sentence structure, coherence, and organization. The AI model, trained on extensive language data, identified errors, offered suggestions for improvement, and provided contextualized recommendations to enhance their writing skills.

ChatGPT engaged participants in a conversational manner, allowing them to ask questions, seek clarification, or request further examples and explanations. The aim was to create an interactive and personalized learning experience that adapted to the individual needs and writing styles of the participants. ChatGPT also offered writing suggestions, alternative phrasing options, and vocabulary expansion ideas to enhance expressiveness and language fluency.

The AI-powered platform included features to track participants’ progress, such as a writing portfolio where completed tasks could be stored and reviewed. Participants were encouraged to reflect on their strengths and weaknesses, incorporate the feedback provided by ChatGPT into their writing revisions, and use the platform’s tools to track their progress. The AI-assisted writing instruction sessions using ChatGPT were conducted twice a week over a period of 12 weeks. Each session lasted for approximately 60 min.

To mitigate plagiarism risks, stringent measures were adopted. The participants received comprehensive guidance on utilizing ChatGPT as a writing aid, emphasizing the creation of original content over reliance on AI-generated material. Engaging in interactive sessions, discussions, and illustrative examples, participants adeptly incorporated AI suggestions while ensuring the authenticity of their work. A considerable emphasis was placed on the ethical use of AI in academic writing, guiding participants on seamlessly integrating AI feedback while preserving their distinctive writing style and thoughts. These measures were designed to empower participants in leveraging AI support while upholding academic integrity. Furthermore, regular interactive discussions actively reinforced these ethical considerations and addressed participant queries throughout the intervention.

#### 3.3.2 Control group

The control group, on the other hand, received traditional writing instruction from an experienced male teacher who used the same instructional materials as the experimental group. Participants attended in-person writing classes led by the teacher.

During the three-month intervention period, control group participants engaged in writing exercises and activities that included a combination of classroom exercises and topics of interest, similar to the experimental group. These activities focused on various aspects of writing, such as grammar, vocabulary, organization, coherence, and sentence structure. The teacher provided individualized feedback on writing assignments, highlighting areas for improvement and offering suggestions for enhancement. Control group participants received feedback exclusively in the classroom during the teacher-led writing classes, which were held regularly, ensuring that participants received ongoing guidance and feedback throughout the 12-week intervention. The amount of time devoted to outside-class practice was designed to be comparable to that of the experimental group.

Unlike the experimental group, the control group did not receive AI-assisted feedback from ChatGPT. Instead, their feedback was based on the teacher’s expertise and teaching experience. The teacher emphasized the importance of practice, guided participants on effective writing strategies, and offered constructive criticism to help develop their writing skills.

Throughout the intervention period, control group participants attended regular writing classes, completed writing assignments, and received feedback from the teacher. They were encouraged to reflect on their writing progress and make necessary revisions based on the feedback provided by the teacher.

The instruction sessions of both groups were held in a dedicated computer laboratory equipped with the necessary technology and internet access. This setting ensured a controlled and consistent environment for both the experimental and control groups. Participants from both groups attended these sessions at the same location, ensuring equitable access to resources.

It is also worth noting that the equivalence of time spent on out-of-class practice between the experimental and control groups was meticulously maintained through the implementation of a time log system. In this system, participants in both groups were tasked with maintaining detailed records of their writing practice beyond their scheduled class sessions. These comprehensive time logs captured essential information, including the duration of each writing session, the specific activities undertaken, and whether the participants utilized ChatGPT (experimental group) or engaged in traditional writing assignments (control group).

The systematic scrutiny of these time logs served as a robust mechanism to affirm the comparability of out-of-class writing practice time between the two groups. This rigorous monitoring and verification process unequivocally ensured that both groups had equitable opportunities for practice and improvement. Table 1 indicates the summary of the intervention details for both groups.

**Table 1:** Summary of the intervention details.

Aspect	Experimental group (AI-assisted)	Control group (Traditional)
Duration	12 weeks	12 weeks
Learning environment	Flexible (home and classroom)	In-person classroom
Writing tasks	Classroom exercises + topics of interest	Classroom exercises + topics of interest
Feedback	AI-powered (ChatGPT)	Teacher
Focus areas	Grammar, vocabulary, organization, coherence, sentence structure	Grammar, vocabulary, organization, coherence, sentence structure
Interactive learning	Yes (with ChatGPT)	Yes (teacher-led classes)
Progress tracking	Yes (writing portfolio)	Yes (writing portfolio)

Following the completion of the 12-week intervention period, all participants, both in the control and experimental groups, underwent a post-test. In line with our commitment to maintaining the comparability of assessments, the post-test featured the same writing tasks, Task 1 and Task 2, extracted from the IELTS writing assessment. Participants were tasked with responding to these writing prompts to gage their progress in academic writing skills and motivation over the course of the study.

### 3.4 Data analysis

In our data analysis, we employed a one-way analysis of covariance (ANCOVA) as the primary method, following the guidance of Pallant (2013). ANCOVA was chosen to assess the impact of two distinct interventions by measuring participants’ outcomes before and after exposure to each intervention, a common approach in pre-test and post-test designs. The independent variable under consideration was the type of intervention, distinguishing between the experimental and control classroom settings. Our dependent variables encompassed global writing performance and specific writing skills, including writing content, organization, and language use, as well as writing motivation.

To enhance the rigor of our analysis and control for any potential initial group differences, we included covariates based on pre-test scores. Additionally, we conducted independent samples t-tests to provide supplementary insights into specific outcome measures, aiming to offer a comprehensive perspective on the effectiveness of the intervention. Furthermore, effect sizes were calculated to quantify the magnitude of observed differences, providing valuable information about the practical significance of our findings. This dual-method approach contributes to a well-rounded and thorough understanding of the intervention’s impact, allowing us to draw more robust conclusions.

Thematic analysis, following the framework outlined by Boyatzis (1998), was employed to scrutinize the qualitative data derived from semi-structured interviews. This rigorous analytical process ensured a systematic exploration of participants’ perspectives on AI-assisted instruction.

Initially, the transcribed interviews underwent open thematic coding. This involved the identification of key variables associated with participants’ experiences and perceptions of engaging with AI-assisted writing instruction. Subsequently, axial coding was implemented to discern intricate relationships among these core variables. This method facilitated the formation of thematic clusters, each encapsulating shared patterns and insights.

The analytical approach adopted in this study was inherently inductive and data-driven, allowing for the emergence of meaningful themes without imposing preconceived notions. To bolster the rigor of the analysis, inter-rater reliability was applied. An experienced EFL researcher, proficient in coding and labeling, collaborated in the validation of the processes. This collaborative effort not only enhanced objectivity but also served to mitigate potential bias.

Throughout this iterative process, adjustments were made to the codification, categorization, and labeling to ensure the robustness and coherence of the identified themes. This comprehensive data analysis framework facilitated a nuanced exploration of participants’ perceptions, contributing valuable insights to the overall findings of this study.

## 4 Results

### 4.1 Quantitative results

Initially, an examination was conducted on the descriptive statistics of the participants’ pre-test and post-test results in overall writing, writing proficiency, and writing motivation across both groups. The summarized findings are presented in Table 2.

**Table 2:** Descriptive statistics for pre-and post-tests scores.

		Pre-test		Post-test	
	Groups	*M*	*SD*	*M*	*SD*
Overall writing	Experimental	39.26	12.03	59.12	14.23
	Control	37.31	17.26	45.18	15.62
Content	Experimental	13.39	3.62	15.96	3.71
	Control	13.26	3.24	13.71	3.12
Organization	Experimental	12.01	3.71	16.56	3.54
	Control	12.34	3.60	13.63	3.63
Language use	Experimental	13.63	3.49	19.89	4.82
	Control	13.51	3.81	15.89	4.12
Writing motivation	Experimental	17.36	3.12	20.06	3.33
	Control	17.71	3.09	18.21	3.58

Table 1 presents descriptive statistics for the pre-and post-test scores in both the experimental and control groups. The mean pre-test score for overall writing was 39.26 (SD = 12.03) in the experimental group and 37.31 (SD = 17.26) in the control group. Following the intervention, the mean post-test score increased to 59.12 (SD = 14.23) in the experimental group and 45.18 (SD = 15.62) in the control group.

For writing content, the experimental group had a mean pre-test score of 13.39 (SD = 3.62) and a mean post-test score of 15.96 (SD = 3.71). In comparison, the control group had a mean pre-test score of 13.26 (SD = 3.24) and a mean post-test score of 13.71 (SD = 3.12). In terms of writing organization, the experimental group had a mean pre-test score of 12.01 (SD = 3.71) and a mean post-test score of 16.56 (SD = 3.54), while the control group had a mean pre-test score of 12.34 (SD = 3.60) and a mean post-test score of 13.63 (SD = 3.63).

Regarding language use, the experimental group had a mean pre-test score of 13.63 (SD = 3.49) and a mean post-test score of 19.89 (SD = 4.82). On the other hand, the control group had a mean pre-test score of 13.51 (SD = 3.81) and a mean post-test score of 15.89 (SD = 4.12). In terms of writing motivation, the experimental group had a mean pre-test score of 17.36 (SD = 3.12) and a mean post-test score of 20.06 (SD = 3.33), while the control group had a mean pre-test score of 17.71 (SD = 3.09) and a mean post-test score of 18.21 (SD = 3.58). The results indicate that the pattern of increase in scores was higher for the experimental group compared to the control group.

The first research question aimed to investigate the impact of AI-assisted instruction on EFL global writing performance. To address this question, we first conducted independent samples t-tests to compare the differences between the experimental and control groups in various writing proficiency components. Additionally, effect sizes were computed to gage the magnitude of these differences.

In overall writing proficiency, a significant difference emerged between the experimental (*M* = 59.12, SD = 14.23) and control groups (*M* = 45.18, SD = 15.62), *t*(48) = 3.79, *p* < 0.001, with a large effect size (Cohen’s *d* = 0.76). For writing content, the experimental group (*M* = 15.96, SD = 3.71) outperformed the control group (*M* = 13.71, SD = 3.12), *t*(48) = 3.01, *p* = 0.003, showing a medium to large effect size (Cohen’s *d* = 0.65).

In writing organization, the experimental group (*M* = 16.56, SD = 3.54) significantly surpassed the control group (*M* = 13.63, SD = 3.63), *t*(48) = 4.12, *p* < 0.001, with a large effect size (Cohen’s *d* = 0.84). Regarding language use, the experimental group (*M* = 19.89, SD = 4.82) demonstrated a substantial advantage over the control group (*M* = 15.89, SD = 4.12), *t*(48) = 4.34, *p* < 0.001, with a large effect size (Cohen’s *d* = 0.88). In terms of writing motivation, the experimental group (*M* = 20.06, SD = 3.33) exhibited significantly higher scores compared to the control group (*M* = 18.21, SD = 3.58), *t*(48) = 3.34, *p* = 0.001, with a medium effect size (Cohen’s *d* = 0.52).

The independent samples t-tests revealed significant differences between the experimental and control groups across various writing proficiency dimensions, with effect sizes highlighting the practical significance of these distinctions.

After that, a one-way ANCOVA was conducted, examining the differences between the experimental and control classrooms and their effects on the post-test scores of students’ writing tests while controlling for the pre-test scores as a covariate in the analysis. Before conducting the ANCOVA, preliminary assessments were carried out to verify the fulfillment of key assumptions. These included examining normal distribution, linearity, equality of variances, homogeneity of regression slopes, and the reliability of covariate measurement. The results of the ANCOVA for the two groups’ global writing performance are presented in Table 3.

**Table 3:** ANCOVA results for global writing.

Source	Type III sum of squares	df	Mean square	*F*	Sig.	Partial eta squared
Group	1237.16	1	1237.16	19.01	0.00	0.27

After controlling for the pre-test scores of writing, statistically significant differences were found between the post-test scores of global writing in the two groups [*F* (1, 47) = 19.01, *p* = 0.00, partial eta squared = 0.27]. The ANCOVA results in Table 3 indicate significant disparities in the improvement of EFL students’ global writing between the experimental and control classrooms. Furthermore, the experimental classroom exhibited higher levels of global EFL writing compared to the conventional classroom.

To further explore the development of EFL students’ writing skills, three additional ANCOVAs were conducted to examine the differences between the experimental and control classrooms in writing content, writing organization, and language use. The ANCOVA results for the two groups’ writing content are presented in Table 4.

**Table 4:** ANCOVA results for content.

Source	Type III sum of squares	df	Mean square	*F*	Sig.	Partial eta squared
Group	9531.62	1	9531.62	12.61	0.00	0.19

According to the findings presented in Table 4, there were statistically significant differences observed between the two groups in terms of writing content [*F*(1, 47) = 12.61, *p* = 0.00, partial eta squared = 0.19]. These results indicate that the experimental classroom significantly outperformed the control classroom in developing EFL students’ writing content.

Also, the ANCOVA results for the two groups’ writing organization are displayed in Table 4. Significant disparities between the two groups in terms of writing organization were observed, as indicated by the results presented in Table 5 [*F*(1, 47) = 23.16, *p* = 0.00, partial eta squared = 0.34]. These findings confirm significant differences between the experimental and control classrooms in enhancing the writing organization skills of the EFL students.

**Table 5:** ANCOVA results for organization.

Source	Type III sum of squares	df	Mean square	*F*	Sig.	Partial eta squared
Group	179.19	1	179.19	23.16	0.00	0.34

Furthermore, the ANCOVA results for the two groups’ language use are depicted in Table 6.

**Table 6:** ANCOVA results for language use.

Source	Type III sum of squares	*df*	Mean square	*F*	Sig.	Partial eta squared
Group	175.22	1	175.22	9.86	0.01	0.11

Table 5 reveals significant distinctions between the two groups in terms of language use [*F*(1, 47) = 9.86, *p* = 0.01, partial eta squared = 0.11]. These findings provide further evidence of significant difference between the experimental and control classrooms in enhancing the language use skills of the EFL students.

The second research question aimed to investigate the impact of the experimental and conventional writing courses on the EFL students’ writing motivation. To examine this, a one-way ANCOVA was employed to compare the post-test scores of writing motivation between the AI-assisted and conventional classrooms. The ANCOVA results for the two groups’ writing motivation are presented in Table 7. Significant differences were observed in writing motivation between the two groups, as presented in Table 7 [*F*(1, 47) = 29.62, *p* = 0.00, partial eta squared = 0.38]. Additionally, it is noteworthy that the experimental classroom exhibited higher levels of EFL writing motivation compared to the conventional classroom.

**Table 7:** ANCOVA results for writing motivation.

Source	Type III sum of squares	df	Mean square	*F*	Sig.	Partial eta squared
Group	89.23	1	89.23	29.62	0.00	0.38

### 4.2 Qualitative results

As for the qualitative phase and to answer the third research question, the thematic analysis of the semi-structured interviews yielded the following themes, providing valuable insights into participants’ experiences, perceptions, and attitudes toward AI-assisted writing instruction.

#### 4.2.1 Theme 1: perceptions of innovation and assistance

Participants expressed positive perceptions of AI-assisted writing instruction. One participant (S1) stated, “I found the AI-assisted writing instruction to be quite innovative and helpful. Having ChatGPT provide real-time feedback on my writing was valuable. It felt like having a personal writing tutor available whenever I needed assistance.” Another participant (S3) mentioned, “At first, I was unsure about using AI for writing instruction. However, as I started using ChatGPT, I realized its benefits. The automated feedback helped me identify and rectify errors in grammar and vocabulary. It was like having an extra set of eyes to improve my writing.” Also, participant S7 shared, “I was surprised by the AI-assisted writing instruction. It was like having a writing coach right there with me. The instant feedback from ChatGPT improved my writing significantly. It was like having a personal guide for my writing learning.”

#### 4.2.2 Theme 2: writing skill enhancements

The implementation of ChatGPT in the writing process yielded remarkable improvements in participants’ writing skills, as articulated in their responses during the interviews. One participant (S6) enthusiastically expressed, “Using ChatGPT improved my writing skills significantly. The instant feedback on organization and coherence helped me structure my essays more effectively. I noticed improvements in my grammar usage and vocabulary selection as well.”

Furthermore, another participant (S9) corroborated this sentiment, emphasizing the transformative impact on their writing prowess, saying, “ChatGPT’s suggestions and examples expanded my vocabulary and improved the flow of my writing. It guided me to express my ideas more clearly and concisely. Overall, my writing became more polished and sophisticated with the help of AI.”

#### 4.2.3 Theme 3: motivation and engagement enhancement

The infusion of AI technology into the writing instruction process led to notable improvements in participants’ motivation and engagement, as revealed in their candid remarks during the interviews. Participant S4 vividly conveyed, “Using AI-assisted writing instruction made writing more enjoyable for me. The interactive nature of ChatGPT and the immediate feedback motivated me to actively engage in the writing process. It increased my motivation to practice and improve my writing skills.” Likewise, participant S10 echoed this sentiment, emphasizing how AI integration heightened their confidence and motivation. They shared, “The integration of AI in writing instruction made me more confident in my writing abilities. Seeing the progress I made with ChatGPT’s guidance boosted my self-efficacy and motivated me to put in more effort to enhance my writing.”

#### 4.2.4 Theme 4: recognized advantages of AI assistance

Participants were keenly attuned to the various advantages offered by AI-assisted writing instruction, and their reflections underscored these benefits. Participant S2 underscored the aspect of accessibility and convenience, sharing, “One advantage of AI-assisted writing instruction is its availability and convenience. I could access ChatGPT anytime and anywhere, which allowed me to practice writing at my own pace and receive immediate feedback.”

Likewise, participant S8 underscored the personalized nature of the AI feedback as a notable advantage, stating, “The personalized feedback provided by ChatGPT was a significant advantage. It addressed my specific writing needs and helped me target areas for improvement. The tailored suggestions and examples were valuable resources for enhancing my writing skills.”

Similarly, participant S6 emphasized the remarkable benefits of personalized feedback, expressing, “The personalized feedback offered by ChatGPT was a game-changer. It pinpointed my unique writing needs, offering tailored suggestions that guided me toward improvement. The inclusion of concrete examples was instrumental in refining my writing skills. It was like having a personal writing coach who understood my growth areas.”

#### 4.2.5 Theme 5: acknowledged challenges and considerations

Although AI-assisted writing instruction was perceived as beneficial, participants acknowledged certain challenges and limitations. One participant (S5) mentioned the contextual accuracy of ChatGPT’s suggestions, stating, “Although AI-assisted writing instruction was beneficial, it had its limitations. Sometimes, ChatGPT’s suggestions were not contextually accurate or aligned with my writing style. It required careful judgment to determine when to accept or modify the AI’s feedback.” Another participant (S11) highlighted the challenge of over-reliance, stating, “One challenge I faced was becoming overly reliant on ChatGPT. I found myself seeking its guidance for every sentence, which hindered my own creativity and critical thinking. It was important to strike a balance between using the AI’s feedback and developing my own writing skills.”

#### 4.2.6 Theme 6: long-term impact and sustainability

Participants also raised considerations regarding the long-term impact and sustainability of AI-assisted writing instruction. One participant (S7) expressed uncertainty about the long-term impact, stating, “I wondered about the long-term impact of AI-assisted writing instruction. While it improved my writing during the study, I questioned if I would continue using AI tools independently after the course. It raised concerns about maintaining the same level of improvement without the AI support.” Another participant (S12) emphasized the need for continuous improvement, stating, “The sustainability of AI-assisted writing instruction is an important aspect to consider. As AI technology evolves, it is crucial to ensure that the AI systems continually improve and adapt to meet the changing needs of language learners. Regular updates and enhancements to AI tools would be beneficial for long-term effectiveness.” Likewise, thinking about the long-lasting effects, participant S14 said, “I thought about how AI-assisted writing instruction affected me in the long run. It definitely made my writing better during the study, but I wasn’t sure if I could keep improving without AI’s help after the course. I was worried about that.”

These qualitative findings provide insights into participants’ perceptions, the impact on writing skills, writing motivation, perceived advantages, challenges, and considerations for the long-term sustainability of AI-assisted writing instruction. The themes and related excerpts from the semi-structured interviews contribute to a comprehensive understanding of the effectiveness of AI-assisted language learning on EFL students’ writing skills and writing motivation in this mixed methods study.

## 5 Discussion

This study sought to delve into the impact of AI-assisted language learning on the academic writing skills and motivation of EFL learners. To provide a comprehensive examination of this phenomenon, a mixed-methods approach was meticulously employed, allowing for the systematic collection and analysis of both quantitative and qualitative data.

The quantitative analysis yielded significant insights, revealing substantial enhancements in the participants’ academic writing performance and writing motivation due to the incorporation of AI-powered language learning. These improvements manifested prominently in the participants’ organizational skills, coherence, grammar, and vocabulary. These quantitative findings resonate with the observations made by Liu et al. (2021) and Yan (2023), who underscored the profound contributions of AI-assisted language learning tools in advancing EFL learners’ writing abilities. Moreover, these findings harmonize with the outcomes identified by Rahman et al. (2022) and Utami and Winarni (2023), which accentuated the positive influence of AI-powered language learning on EFL learners’ motivation and their heightened engagement in writing tasks.

One possible explanation for the aforementioned findings may be attributed to the high level of engagement exhibited by EFL learners in writing activities supported by AI. The AI-assisted language learning tool offered the learners suitable alternatives for their written texts, making this approach more favorable compared to conventional writing instruction (Zhao, 2022). By generating writing ideas, grammatically accurate sentences, and appropriate lexical resources, the AI tool facilitated the production of better-written texts, thereby enhancing the learners’ engagement in the required writing tasks within the AI-supported class. This heightened engagement in writing activities potentially contributed to the development of the learners’ academic writing skills. These findings align with the research of Utami and Winarni (2023), who also found that AI-powered language learning tools improved EFL learners’ engagement in writing activities, consequently leading to improvements in writing skills. The qualitative findings of the current study further corroborated these quantitative findings, as learners exhibited positive responses toward the integration of the AI-assisted language learning approach into their writing activities.

Consistent with the findings of Hwang et al. (2023), the present study suggests that the positive outcomes can be attributed to the personalized language learning experience facilitated by the AI-assisted language learning tool during collaborative writing tasks. By using the AI-powered tool, learners had the opportunity to work at their own pace and receive immediate feedback, enabling them to produce better-written texts. This personalized learning experience not only enhanced learners’ writing motivation but also provided specific feedback and comments on their writing tasks, assisting them in addressing writing issues more effectively and conveniently. These findings align with the qualitative findings of the study, wherein learners expressed their enthusiasm for the AI tool’s ability to personalize their writing activities. This aspect of the AI-assisted language learning tool is also in accordance with Vygotsky’s (1984) social constructivist theory, as it allowed learners to interact with the AI tool and gradually internalize their writing abilities. In other words, the AI tool initially supported learners in personalizing their writing activities, leading to the internalization of writing skills.

Aligned with Vygotsky’s (1984) social constructivist framework, the integration of AI introduced a dynamic incentive for learners to exercise self-regulation in their writing endeavors. Following this theory, participants in this study engaged in collaborative writing tasks with AI, a practice that facilitated the development of their self-regulatory capacities. Through these interactive sessions, learners transitioned from relying on external regulation to becoming adept at independently managing writing tasks. This discovery implies that learners who availed themselves of the AI-assisted language learning approach were not only able to attain writing self-regulation more promptly but also exhibited a higher degree of effectiveness compared to their counterparts who did not incorporate AI into their writing routines. Substantiating this claim, qualitative data from the study underscored the significant role of the AI tool in empowering learners to function autonomously within the AI-powered learning environment, thus corroborating the earlier findings.

One potential explanation for the quantitative findings of this study may lie in the tendency of EFL learners to experience anxiety when engaging in individual or collaborative writing tasks within traditional classroom settings (Yan, 2023). The completion of writing tasks under such traditional circumstances can have a detrimental effect on EFL learners’ writing performance and motivation. In contrast, the integration of the AI-assisted language learning tool in the AI class created a less anxiety-inducing context for learners to practice and enhance their academic writing skills and motivation. This finding aligns with the qualitative results, wherein learners expressed that the AI environment alleviated their writing anxiety and facilitated improvements in their writing abilities.

Furthermore, the quantitative facet of this study lends credence to the proposition that the AI-assisted language learning approach fosters the development of EFL learners’ academic writing skills. Consequently, it is plausible to posit that this approach may also be advantageous for other writing genres, such as creative writing and argumentative writing (Ippolito et al., 2022; Su et al., 2023). Su et al. (2023), for instance, contend that AI-assisted language learning tools exert a positive influence on learners’ argumentative writing by furnishing immediate feedback on language usage, organization, and content. The versatility of AI tools in providing feedback and commentary across various dimensions of writing performance holds the potential to augment the individual writing skills of EFL learners.

The qualitative findings also offer nuanced insights into the impact of AI-assisted writing instruction on EFL learners. The positive perceptions echoed by participants align with prior research, emphasizing the significance of real-time feedback as a pedagogical tool (Zhang and Hyland, 2018). The automated feedback mechanism proved instrumental in addressing nuanced aspects of writing, such as grammar, vocabulary, and overall organization. This is consistent with studies by Barrot (2023) and Zhao (2022), which emphasize how immediate feedback can significantly contribute to enhanced writing proficiency.

Furthermore, participants reported notable improvements in their writing skills attributed to the use of ChatGPT. The intervention’s effectiveness in enhancing organization and coherence within their essays demonstrates the potential of AI-assisted tools in scaffolding the writing process (Zhao, 2022; Barrot, 2023). Additionally, the incorporation of suggestions and examples by ChatGPT resulted in an expanded vocabulary and improved fluency in writing. This mirrors the findings of studies that have highlighted AI’s contribution to vocabulary development and writing fluency (Ippolito et al., 2022; Su et al., 2023).

The qualitative analysis also unveils the positive impact of AI integration on participants’ motivation and engagement, affirming previous studies that have identified similar trends (Huang and Tan, 2023). The interactive nature of ChatGPT, coupled with immediate feedback, fostered a more engaging and enjoyable writing process. Moreover, the confidence boost observed among participants aligns with Su et al.’s (2023) assertion that AI tools positively influence learners’ confidence in their writing abilities.

While the advantages of AI-assisted writing instruction are prominent, participants also acknowledged certain challenges. The identified contextual accuracy concerns resonate with previous studies highlighting the need for careful consideration when incorporating AI-generated feedback (Utami and Winarni, 2023). This cautious approach is crucial to ensure that AI feedback aligns with the individual writing styles and contexts of learners. Additionally, the challenge of over-reliance on ChatGPT emerged as a noteworthy consideration. Striking a balance between utilizing the AI’s feedback and fostering independent critical thinking and creativity is vital (Utami and Winarni, 2023). This echoes the broader discourse on scaffolding in education, emphasizing the need for gradual release of responsibility from the AI tool to the learner. Lastly, participants expressed considerations regarding the long-term impact and sustainability of AI-assisted writing instruction. These concerns align with broader discussions on the evolving role of AI in education. Continuous improvement and adaptability of AI systems to cater to the changing needs of language learners are indeed critical aspects for its sustained effectiveness (Su et al., 2023).

In essence, the qualitative findings not only validate the quantitative results but also provide rich contextual insights into the multifaceted impact of AI-assisted writing instruction on EFL learners. This comprehensive understanding highlights the potential of such interventions in advancing writing skills and motivation among language learners.

## 6 Conclusion

Building upon Vygotsky’s social constructivist framework, the primary aim of this study was to explore the impact of an AI-assisted language learning approach on the academic writing skills and writing motivation of Chinese EFL learners. The quantitative results revealed that the AI-assisted class exhibited superior development and performance in terms of academic writing skills and writing motivation compared to the non-AI class. Complementing the quantitative findings, the qualitative results provided further insights into the EFL learners’ positive perceptions regarding the incorporation of the AI-assisted language learning approach in their writing courses. These perceptions stemmed from the interactive and innovative learning environment facilitated by the AI tool, which played a significant role in enhancing the learners’ academic writing skills and writing motivation.

The findings of this study carry important implications for the field of EFL, particularly in relation to the teaching of writing. The integration of AI tools into EFL writing courses is recommended as part of a technology-integrated language learning approach, as it aligns with innovative pedagogical practices and has the potential to significantly enhance students’ academic writing skills and writing motivation. To enhance EFL learners’ academic writing skills and motivation, it is highly encouraged for learners to actively incorporate AI tools into their writing activities. By leveraging the capabilities of AI, learners can receive immediate and targeted feedback, enabling them to identify areas for improvement and enhance their writing performance accordingly. This integration of AI into their writing practice has the potential to yield more efficient and effective learning outcomes.

To provide engaging and motivating writing activities supported by AI, EFL educators and teachers should consider establishing dedicated AI-supported language learning classes. These classes can serve as platforms to train EFL learners on how to effectively utilize AI tools to improve their writing skills. Through providing guidance and support in the use of AI tools, educators can empower learners to take ownership of their learning process and make meaningful progress in their writing abilities. Moreover, organizing an AI-supported language learning class specifically tailored to writing activities can bring significant benefits to EFL learners. Within such a class, learners can receive AI-generated writing feedback and comments addressing various aspects of their written texts, including language usage, organization, and content. This comprehensive feedback can provide learners with specific guidance to address their writing challenges and refine their skills across different areas.

Several limitations should be acknowledged when interpreting the findings of this study. Firstly, the generalizability of the results may be constrained due to the specific sample of Chinese EFL students that was used. The cultural backgrounds, educational systems, and language proficiency levels in other contexts may differ, potentially yielding different outcomes. Therefore, caution should be exercised in generalizing the findings beyond the specific population studied. Replication studies with more diverse populations are needed to validate the effectiveness of AI-assisted language learning in different settings. Secondly, the study utilized a relatively small sample size of 50 participants, which may have implications for the statistical power and generalizability of the findings. A larger sample size would have provided more robust results and increased the confidence in the conclusions drawn from the study. Future research endeavors should consider recruiting a larger and more diverse participant pool to enhance the external validity of the findings. Another limitation of the study is the relatively short duration of the intervention. The impact of AI-assisted language learning on writing skills and motivation was evaluated over a limited period, potentially limiting our understanding of the sustained effects of such instruction. Longer intervention periods would offer insights into the durability of the observed improvements. Future studies should explore the long-term effects of AI-assisted language learning to provide a more comprehensive assessment of its efficacy in enhancing writing abilities. Furthermore, a potential limitation is the potential for contamination between the experimental and control groups. Despite random assignment, it is challenging to completely separate instructional methods or interactions in real-world educational settings. Some degree of crossover between the groups may have occurred, introducing the possibility of contamination effects and influencing the observed results. Future studies could implement more stringent control measures to minimize contamination and provide a more accurate assessment of the effects of AI-assisted language learning.

Additionally, despite the rigorous implementation of measures to mitigate potential risks, it is imperative to acknowledge certain limitations within our study. One such aspect pertains to the challenge of ensuring the prevention of unintended plagiarism due to the utilization of AI-assisted writing tools. Although extensive guidance was provided to the participants on the ethical use of ChatGPT as a supplementary writing aid, aimed at fostering original content creation and upholding academic integrity, the inherent nature of AI-generated suggestions poses a plausible risk if not meticulously monitored. Although the participants were duly instructed on incorporating AI-generated feedback while preserving their individual writing styles, the potential for unintentional reliance on AI-generated content remains a concern. Further steps, such as continual reinforcement of ethical considerations and vigilance in recognizing potential plagiarism risks, could enhance the robustness of future studies employing AI-assisted writing tools. Therefore, while our study diligently emphasized the importance of academic integrity and ethical AI use, the dynamic nature of AI assistance poses an ongoing challenge in safeguarding against unintended plagiarism. Future research endeavors employing AI tools may benefit from refined methodologies and continuous vigilance to further address this critical concern.

Lastly, the study did not include a long-term follow-up to assess the sustainability of the observed improvements in writing skills and motivation. Without a post-intervention evaluation, it remains unknown whether the benefits of AI-assisted instruction persist over an extended period of time. Future research should consider conducting follow-up evaluations to ascertain the longevity of the observed enhancements and further investigate the potential long-term impact of AI-assisted language learning on writing skills and motivation.

## Data availability statement

The raw data supporting the conclusions of this article will be made available by the authors, without undue reservation. Requests to access these datasets should be directed to CS, songcp2023@163.com.

## Ethics statement

The studies involving humans were approved by the School of Foreign Studies, North Minzu University, Yinchuan, Ningxia. The studies were conducted in accordance with the local legislation and institutional requirements. The participants provided their written informed consent to participate in this study.

## Author contributions

CS: Conceptualization, Data curation, Investigation, Methodology, Project administration, Resources, Validation, Visualization, Writing – original draft, Writing – review & editing. YS: Data curation, Formal analysis, Investigation, Methodology, Project administration, Resources, Software, Visualization, Writing – original draft, Writing – review & editing.
