# Correction: Enhancing academic writing skills and motivation: assessing the efficacy of ChatGPT in AI-assisted language learning for EFL students

**DOI:** 10.3389/fpsyg.2026.1917101

**Published:** 2026-07-22

**Authors:** Cuiping Song, Yanping Song

**Affiliations:** 1 School of Foreign Studies, North Minzu University, Yinchuan, Ningxia, China; 2 School of Public Administration, Central South University, Changsha, Hunan, China

**Keywords:** AI-assisted language learning, writing skills, writing motivation, EFL students, mixed methods study, ChatGPT

The statistical results presented in Table 4 are correct: *F*(1, 47) = 12.61, *p* = 0.00, partial eta squared = 0.19. These values indicate a statistically significant difference between the experimental and control groups in writing content. However, during the final editing and proofreading stage of the manuscript, an error was introduced. The first sentence describing Table 4 was incorrectly left as “there were no statistically significant differences,” while the intended wording—and the wording consistent with the table and the subsequent sentence was “there were statistically significant differences.” The second sentence (“These results indicate that there were a significant difference…”) correctly reflects the significant finding.

A correction has been made to section **4 Results**, *subsection 4.1 Quantitative results*, paragraph 12:

“According to the findings presented in Table 4, there were statistically significant differences observed between the two groups in terms of writing content [*F*(1, 47) = 12.61, *p* = 0.00, partial eta squared = 0.19]. These results indicate that the experimental classroom significantly outperformed the control classroom in developing EFL students' writing content.”

The original version of this article has been updated.

