# Supplementary material for: Enhancing academic writing skills and motivation: assessing the efficacy of ChatGPT in AI-assisted language learning for EFL students
Source: Front Psychol. 2023 Dec 15;14:1260843. doi: 10.3389/fpsyg.2023.1260843 (PMC10754989; doi:10.3389/fpsyg.2023.1260843)
Supplement: Supplementary file 1 [file Table_1.docx]

**Appendix A: Sample IELTS Academic Writing Task and Scoring Criteria**

*Task Description:*

**Writing Task 2: Essay (40 minutes)**

In this task, you are required to write an essay in response to a question or topic. You should present an argument, provide evidence, and reach a conclusion. The essay should be well-structured, with an introduction, body paragraphs, and a conclusion.

**Topic:** Discuss the advantages and disadvantages of online learning compared to traditional classroom learning. Include relevant examples and your own experiences.

*Scoring Criteria:*

The following scoring criteria will be used to evaluate your essay:

1. **Task Achievement (TA)**
   - Addresses all aspects of the task.
   - Presents a clear and well-defined position on the topic.
   - Provides relevant examples and evidence to support arguments.
2. **Coherence and Cohesion (CC)**
   - Organizes ideas logically, with clear paragraph structure.
   - Uses cohesive devices (e.g., linking words, pronouns) to connect ideas.
   - Maintains a clear and consistent overall structure (introduction, body, conclusion).
3. **Lexical Resource (LR)**
   - Demonstrates a wide range of vocabulary.
   - Uses vocabulary appropriately and effectively.
   - Avoids overuse of repetitive language.
4. **Grammatical Range and Accuracy (GRA)**
   - Utilizes a variety of sentence structures.
   - Demonstrates accurate grammar and sentence construction.
   - Minimizes errors in grammar and punctuation.

***Scoring Rubric****:*

Each criterion will be scored on a scale from 0 to 9, and then the scores for each criterion will be averaged to determine the overall score for your essay.

- 9-8: Very Good
- 7-6: Good
- 5: Satisfactory
- 4: Limited
- 3: Partial
- 2: Extremely Limited
- 1: Intermittent
- 0: Did not attempt the task

Remember that your essay will be assessed based on these criteria, and the final score will reflect your overall performance in addressing the task, coherence and cohesion, lexical resource, and grammatical range and accuracy. Good luck with your writing!

**Appendix B:** *Writing Motivation Scale (WMS) Items*

I enjoy writing in English.

Writing in English is very important to me.

I always look forward to my ESL writing classes.

I would like to spend lots of time learning to write in English.

I would like to concentrate on learning to write in English more than any other topic.

I actively think about what I have learned in my English writing class.

I really try to learn how to write in English.

**Appendix C:** *Interview Questions*

1. Can you describe your overall experience with AI-assisted writing instruction using ChatGPT?
2. In what specific ways did you notice improvements in your writing skills while using ChatGPT?
3. How did the integration of AI in writing instruction affect your motivation and engagement in writing tasks?
4. Can you share any advantages or benefits you experienced with AI-assisted writing instruction?
5. Were there any challenges or limitations you encountered when using ChatGPT for writing instruction?

**Appendix D:** *Examples of Feedback - Teacher and AI*

*Teacher Feedback:*

**Participant: Student 1**

*Essay Excerpt:* "The benefits of online learning are undeniable. It offers flexibility and convenience for learners. However, traditional classroom learning also has its merits."

*Teacher Feedback:*

- "Your introduction is clear and sets up the topic effectively."
- "You've provided a good overview of the advantages of online learning, but remember to include counterarguments."
- "Consider using transition words to improve the flow between your ideas."
- "Watch out for subject-verb agreement errors. For example, 'benefits' should agree with 'are'."

**Participant: Student 2**

*Essay Excerpt:* "I believe that online learning is superior to traditional classroom learning in many ways. Firstly, it allows students to access course materials at any time."

*Teacher Feedback:*

- "Your position is clear, but remember to address potential disadvantages as well."
- "You've made a strong point about accessibility in online learning."
- "Work on varying your sentence structures for better fluency."
- "Check for singular/plural agreement in 'students' and 'materials'."

*AI Feedback:*

**Participant: Student 1**

*Essay Excerpt:* "The advantages of online learning are numerous. It offers flexibility for learners."

*AI Feedback:*

- "Well done on highlighting the advantages. Now, consider elaborating on each point for a more comprehensive argument."
- "Try using transition words like 'Firstly,' to make your points clearer."
- "Your sentence structure is clear, but try varying it to keep the reader engaged."

**Participant: Student 2**

*Essay Excerpt:* "I strongly believe that online learning is far superior to traditional classroom learning."

*AI Feedback:*

- "Your stance is evident, but remember to discuss both sides of the argument for a balanced essay."
- "You've made a convincing point about superiority. Can you provide evidence to support your claim?"
- "Your sentence structure is good, but consider adding more complex sentences for variety."

These examples illustrate the type of feedback provided to participants in both the control group (teacher feedback) and the experimental group (AI feedback) during the study. The feedback aims to address various aspects of writing, including argument development, organization, sentence structure, and grammar.
